# Lichenoid areas may arise in early stages of proliferative verrucous leukoplakia: A long‐term study of 34 patients

**DOI:** 10.1111/jop.13317

**Published:** 2022-06-03

**Authors:** Catalina Barba‐Montero, Alejandro Ismael Lorenzo‐Pouso, Pilar Gándara‐Vila, Andrés Blanco‐Carrión, Xabier Marichalar‐Mendía, Abel García‐García, Mario Pérez‐Sayáns

**Affiliations:** ^1^ Oral Medicine, Oral Surgery and Implantology Unit, MedOralRes Group University of Santiago de Compostela Santiago Spain; ^2^ ORALRES Group Health Research Institute of Santiago de Compostela (IDIS) Santiago Spain; ^3^ Department of Nursing, Faculty of Medicine and Nursing University of Basque Country Leioa Spain

**Keywords:** dysplasia, malignant transformation, oral lichen planus, oral lichenoid lesions, proliferative verrucous leukoplakia

## Abstract

**Background:**

Proliferative verrucous leukoplakia is considered an uncommon oral potentially malignant disorder with a high malignant transformation rate. The objective of this paper was to define its cancer incidence and related risk factors.

**Methods:**

A retrospective audit of 34 patients diagnosed with proliferative verrucous leukoplakia from a university‐based unit, during the period from 1995 to 2019 was performed. The mean number of visits was 23 ± 18.6. The follow‐up was divided into four‐time intervals to evaluate the clinical presentation, number of lesions, dysplasia grade, and malignant transformation rate.

**Results:**

The majority of patients were females 29 (85.3%), with verrucous component (77.8%), with a gingival presentation (31.8%), and with a preceding lichenoid area (44.1%). Eleven patients (32.4%) were affected by oral cancer during the follow‐up, developing a total of 15 carcinomas. The mean age of malignant transformation was 67.2 ± 12.9 years, particularly 8 ± 8.5 from the onset of the lesions. Warty forms presented a higher mean estimate for malignant transformation (15.2 years, 95% confidence interval 4.4–26 years) than nodular forms (1.9 years, 95% confidence interval 1.9–1.9) (*p* = 0.019). Patients with an initial proliferative verrucous leukoplakia diagnosis suffered a higher risk of malignancy, particularly 15.55 times (95% confidence interval 1.69–143.17; *p* = 0.015) than those who did present a preceding area with lichenoid morphology.

**Conclusion:**

Proliferative verrucous leukoplakia presented a high malignant transformation rate and sometimes displayed preceding oral lichenoid areas in early stages. Further studies are needed to understand the impact of these lichenoid areas in proliferative verrucous leukoplakia progression.

## 1 INTRODUCTION

Oral leukoplakia (OL) is considered the most prevalent and significant oral potentially malignant disorders (OPMDs) worldwide. OL is defined as “a predominantly white plaque of questionable risk having excluded (other) known diseases or disorders that carry no increased risk for cancer.”^1^, ^2^ The last World Health Organization (WHO) Collaborating Centre for Oral Cancer position paper regarding OPMDs nomenclature found no logical reason to change this classical definition. Nonetheless, this working group decided to define separately classical OL from a particular form of this disorder, namely proliferative verrucous leukoplakia (PVL).^3^ The term PVL was first coined more than 35 years ago by Hansen et al.^4^ Since then, individual reports, small case series, and reviews have been published regarding this enigmatic form of OL.^5^ Despite these continuous efforts, PVL diagnosis criteria remained unresolved for decades. Indeed, even the aetiopathogenesis and associated risk factors of these OPMDs remain unclear. In order to unify reporting guidelines, this latest WHO working group ended up defining PVL as a “progressive, persistent, and irreversible disorder characterized by the presence of multiple leukoplakias that frequently become warty.”^3^

It is worth mentioning that PVL does not carry a histopathologic connotation but subtle pathologic features, such as the presence of lichenoid chronic inflammation, dense/wavy hyperkeratosis, acanthosis, papillomatous squamous proliferation, and variable degrees of dysplasia.^6^ Moreover, this clinical entity is well known for its marked tendency to recur. In this sense, a recent meta‐analysis yielded a pooled recurrence rate of 67.2%.^7^ The most important complication of PVL is the development of oral cancer.^8^ This fact carries relevant implications for the management of these patients, especially in regard to the surveillance programs that they must receive, given that these disorders are likely to accompany them during all their lives.^2^ The malignant transformation (MT) rate of this OPMD is among the highest of its spectrum ranging from 43.8% to 65.8%.^9^, ^10^ In addition, several authors have considered that field cancerization exerts a relevant effect on this disorder leading frequently to multiple primary tumors in these patients.^11^ Taking together, the crucial point for PVL diagnosis and management is the keen observation of its gradual topographical, and histopathological changes.^12^

In the authors' experience, we consider the proliferative or multifocal nature of the disease as the main cornerstone for diagnosis.^9^ Other researchers consider the presence of verrucous features as a main diagnostic criteria, or even the same authors consider all gingival leukoplakias regardless of size to be PVLs.^13^ These factors for establishing a PVL diagnosis case may be over‐restrictive and even confusing, implying a potential underdiagnosis and misestimation of the true malignant transformation rate of this uncommon but relevant OPMD.

Prompted by discussed literature, we undertook a retrospective study of a carefully documented case series to elucidate any early features or risk factors noted in clinical or histopathological records. The objective of the study was twofold: (i) to describe the clinical and histopathological features of the lesions during the follow‐up; and (ii) to investigate the cancer incidence in patients with this OPMD and its associated risk factors.

## 2 MATERIAL AND METHODS

### 2.1 Study population

The study is a retrospective audit of patients from a university‐based unit diagnosed with PVL. Ethical clearance for the study was obtained from the Ethical Review Committee of the Faculty of Medicine and Dentistry at the University of Santiago de Compostela (Ref. 2016/337). Patient records were searched to identify cases of PVL and then cross‐matched with oral cancer records from Galician Health Service (SERGAS) Cancer Registry data up until the end of 2019.^14^

Thirty‐four patients with a clinical diagnosis of PVL (based on WHO Collaborating Centre for Oral Cancer criteria—see^3^) confirmed with a biopsy and managed during the period from 1995 to 2019 were selected for inclusion. Out of a preliminary selection, 12 patients were excluded due to a lack of complete clinical information.

Initially, all patients were biopsied to establish diagnoses. Particularly, we used an incisional technique involving the removal of a representative portion of the target white patch but also a part of adjacent healthy tissue. Due to the multifocal nature of PVL, different samples in each intervention were obtained, placing each of them in a separate and adequately identified container for further histopathological analysis. During follow‐up, we opt to perform biopsies upon the detection of an oral anomaly (e.g., new ulceration, tumor, or tissue growth in mucosa). Selected regions were chosen on the basis of a conventional oral examination performed by two trained specialists in oral medicine (ABC and PGV) using 2.5/3 magnification loupes with white LED headlight, particularly BLD‐3 (Neitz Instruments Co., Ltd.). The samples were fixed in formaldehyde and were subjected to a histopathological evaluation. Pathology reporting on oral epithelial dysplasia for this case‐series was presented according to binary and the WHO 2005 histologic grading systems.^15^, ^16^ This analysis was performed by senior oral pathologists calibrated to each other, as previously reported.^17^

All patients were informed that they had an OPMD, stressing the high frequency of malignant transformation and allowing them sufficient time to raise all their doubts or questions. Patients were advised of the need for regular follow‐ups over their lifetime, given the unpredictability of oral cancer development. Follow‐ups were programmed 2 months after initial diagnosis. Patients were instructed to contact the clinician between visits immediately upon the detection of an oral anomaly or suspicion also to clarify any doubt that might arise. All patients who missed a follow‐up session were contacted by phone.

The mean number of visits was 23 ± 18.6. In order to tackle the problem of multiple periods of follow‐up among patients, this period was divided into time intervals. Globally, follow‐up was divided into four intervals segmented as follows: T0 the initial visit with a histopathological assessment, T1 ¼ of the follow‐up, T2 ¾ of the follow‐up, and TF the last recorded appointment.

In the event of a malignant transformation, it was collected at the closest corresponding interval. WHO malignant transformation definition (i.e., biopsy‐proven PVL) was used in this audit, including only those cases of PVL that have had their diagnosis confirmed by both clinical examination/photographs and histological assessment prior to malignancy. Information on the age at diagnosis, gender, presence of lichenoid areas, unhealthy oral habits, systemic health status, location, number of lesions, date of diagnosis, and treatment for PVL such as cold knife excision, or laser therapy was collected from clinical charts. A pre‐tested questionnaire was posted to all participants who were on follow‐up in order to avoid data attrition.

### 2.2 Statistical analysis

The results were analyzed using the SPSS Statistics 20.0 (SPSS Inc.) for Mac (SPSS Inc.) by an investigator who was blinded to the type of interventions analyzed. A descriptive analysis of clinical and pathological factors was carried out. Student's *t* test or the analysis of variance single‐factor test was applied to compare means. To assess the association between risk factors and malignant transformation, we transformed the variables into binary combinations and used the Pearson chi‐square test or Fisher's exact test. We also developed a logistic regression analysis to assess the effect of some risk factors. For survival analysis, Kaplan–Meier and log‐rank tests were used. The study time was considered the time point from the completion of the first biopsy to the development of cancer (non‐censured observation) or until the end of the patient follow‐up (censored observation). p Value <0.05 was considered as significant.

## 3 RESULTS

### 3.1 Characteristics of study population and clinical presentation

**FIGURE 1:**
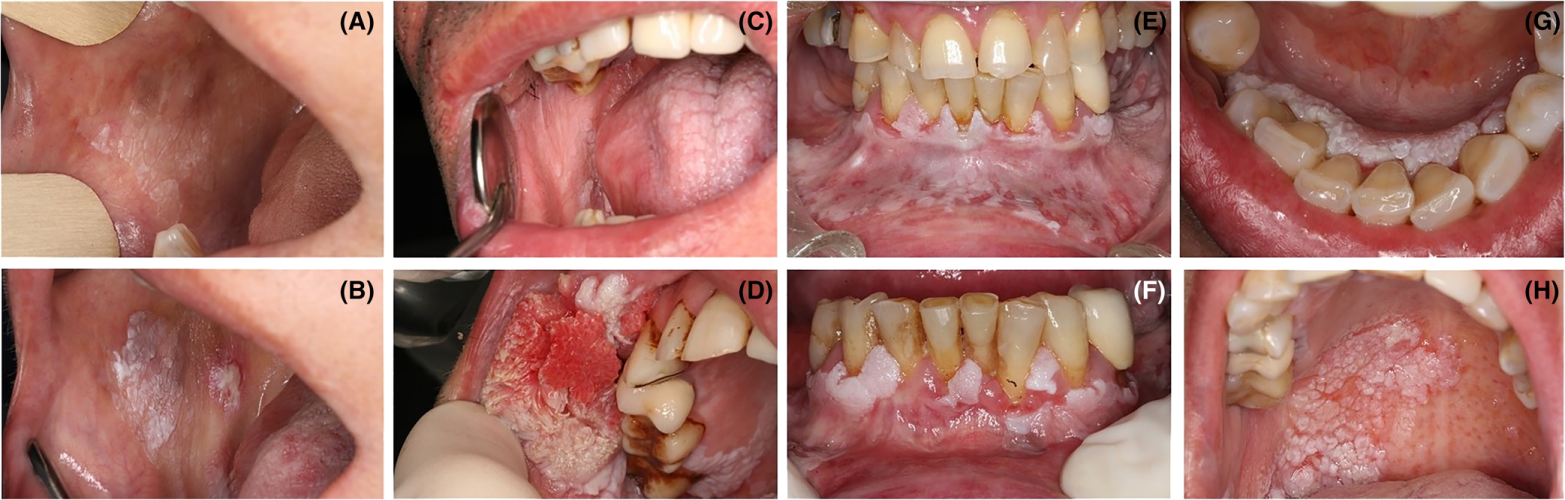
Representative cases of patients affected by proliferative verrucous leukoplakia. (A, B) Patient with a widespread non‐homogenous leukoplakia of the buccal mucosa that ended up in a verrucous carcinoma. (C, D) Patient with a widespread homogenous gingival and palatal leukoplakia that progressed to a conventional squamous cell carcinoma. (E, F) Patient affected by a gingival proliferative verrucous leukoplakia treated with laser vaporization. Images in (G, H) present a patient with an initial gingival leukoplakia that underwent lately a conventional squamous cell carcinoma of the hard palate.

A total of 34 patients were eligible for the study with a complete data set (Figure 1). Table 1 shows the demographic and clinical features of patients diagnosed with PVL. The majority of patients were females 29 (85.3%), whilst 5 (14.7%) were males. The mean age of patients at the onset of lesions was 56 ± 16.6 years and the mean age of the PVL formal diagnosis was 62.4 ± 12.1. Table 2 presents all the time intervals collected in the study. In terms of tobacco use, 85.3% were non‐smokers and 88.2% non‐drinkers. The most common clinical form was the non‐homogeneous (52.9%) and specifically, 77.8% of the patients had a verrucous component. It should be noted that, in 44.1% of the cases, displayed a lichenoid area. PVLs were treated with conventional surgery in 73.5% of the cohort and CO^2^ laser vaporization in the remaining 26.5%.

**TABLE 1:** Demographic characteristics and clinical features of the study population

Systemic diseases	Yes *N* (%)	No *N* (%)	Total *N* (%)
Gender			
Men	4 (11.8%)	1 (2.9%)	5 (14.7%)
Women	19 (55.9%)	10 (29.5%)	29 (85.3%)
Total	23 (67.7%)	11 (32.3%)	34 (100%)

Psychiatric diseases	Yes *N* (%)	No *N* (%)	Total *N* (%)
Gender			
Men	0	5 (14.7%)	5 (14.7%)
Women	5 (14.7%)	24 (70.5%)	29 (85.3%)
Total	5 (14.7%)	29 (85.3%)	34 (100%)

Polypharmacy	Yes *N* (%)	No *N* (%)	Total *N* (%)
Gender			
Men	1 (2.9%)	4 (11.8%)	5 (14.7%)
Women	4 (11.8%)	25 (73.5%)	29 (85.3%)
Total	5 (14.7%)	29 (85.3%)	34 (100%)

Death	Yes *N* (%)	No *N* (%)	Total *N* (%)
Gender			
Men	0	5 (14.7%)	5 (14.7%)
Women	0	29 (58.3%)	29 (58.3%)
Total	0	34 (100%)	34 (100%)

Relapse in the treated zone	Yes *N* (%)	No *N* (%)	Total *N* (%)
Gender			
Men	2 (5.8%)	3 (8.8)	5 (14.7%)
Women	6 (17.6%)	23 (67.7%)	29 (58.3%)
Total	8 (23.5%)	26 (76.4%)	34 (100%)

Synergist with oral lichen planus/lichenoid lesions	Yes *N* (%)	No *N* (%)	Total *N* (%)
Gender			
Men	2 (5.8%)	3 (8.8%)	5 (14.7%)
Women	13 (38.2%)	16 (47.1%)	29 (58.3%)
Total	15 (44.1%)	19 (55.9%)	34 (100%)

Tobacco consumption	Non‐smoker *N* (%)	Former smoker *N* (%)	Current smoker *N* (%)	Total *N* (%)
Gender				
Men	3 (8.8%)	1 (3%)	1 (3%)	5 (14.7%)
Women	20 (58.8%)	6 (17.5%)	3 (8.8%)	29 (58.3%)
Total	23 (67.6%)	7 (20.5%)	4 (11.8%)	34 (100%)

Alcohol consumption	No drinker *N* (%)	<1 SDU *N* (%)	>1 SDU *N* (%)	Total *N* (%)
Gender				
Men	3 (8.8%)	1 (3.0%)	1 (3%)	5 (14.7%)
Women	27 (79.5%)	2 (5.8%)	0	29 (85.3%)
Total	30 88.2%)	3 (8.8%)	1 (3%)	34 (100%)

Clinical form of PVL	Homogeneous *N* (%)	Non‐homogeneous *N* (%)	Total *N* (%)
Gender			
Men	0	5 (14.7%)	5 (14.7%)
Women	16 (47.1%)	13 (38.2%)	29 (85.3%)
Total	16 (47.1%)	18 (52.9%)	34 (100%)

Non‐homogenous clinical form	Verrucous *N* (%)	Nodular *N* (%)	Erythro‐leukoplakia *N* (%)	Total *N* (%)
Gender				
Men	5 (14.7%)	0	0	5 (14.7%)
Women	9 (26.4%)	2 (5.8%)	2 (5.8%)	29 (85.3%)
Total	14 (41.2%)	2 (5.8%)	2 (5.8%)	34 (100%)

Abbreviation: SDU, standard drink unit.

**TABLE 2:** Time intervals and clinical presentation of patients of diagnosed with proliferative verrucous leukoplakia stratified by outcome

	*N*	Average ± SD	Minimum	Maximum
Age of onset of the lesions	34	56 ± 16.6	15.2	79.8
Age of formal diagnosis of VPL	34	62.4 ± 12.1	35.7	81.2
Age of malignancy	11	67.2 ± 12.9	48.5	82.2
Time to malignancy from onset of lesions	11	8 ± 8.5	0.5	28.5
Time to malignancy from diagnosis	11	1.7 ± 2.1	0.0	6.1
Average follow‐up time	34	5.7 ± 4.1	0.6	14.3
T0 total number of lesions		9.4 ± 5.0	2	20
T0 total number of locations		4.8 ± 2.4	1	10
T1 total number of lesions		10.4 ± 5.1	3	21
T1 total number of locations		5.1 ± 2.3	1	9
T2 total number of lesions		10.8 ± 5.5	2	24
T2 total number of locations		5.4 ± 2.4	1	10
TF total number of lesions		9.6 ± 5.1	2	23
TF total number of locations		5 ± 2.2	1	10

**FIGURE 2:**
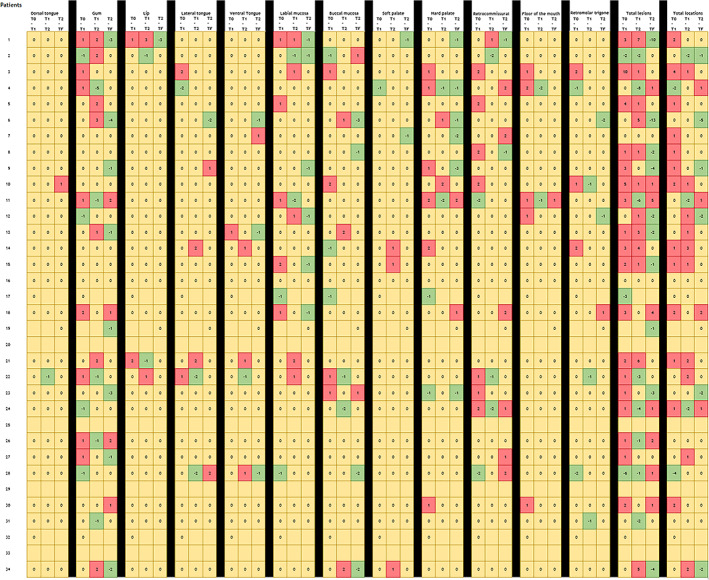
Heat‐map‐like diagram representing the increase/decrease of number of lesions in each affected site for patient, according to the location and the time intervals. Each number across tables reflects the number of lesions during the transition of time intervals and studied regions (columns) in the cohort of patients (rows). In terms of colors: red implies an increase in the number of lesions of the studied area, yellow implies a static clinical behavior, and green a reversal in the number of affected regions. Time intervals were segmented as follows: T0 the initial visit with histopathological assessment, T1 ¼ of the follow‐up, T2 ¾ of the follow‐up, and TF the last recorded appointment.

Considering the four intervals of follow‐up, gingiva was the most frequent location (31.8%), followed by the buccal mucosa (30.4%). Regarding the number of affected areas, they are extensively displayed in Figure 2 as a heat‐map. The larger the follow‐up, the more pronounced number of affected regions and higher number of lesions. Paradoxically, treatment modality did not exhibit an effect on these outcomes. The global proportion rate for recurrence was 23.5% with no significant differences between both arms of therapies. None of the participants died as a consequence of the disease at the end of the follow‐up.

### 3.2 Rate of malignant transformation and associated factors

The mean follow‐up time from diagnosis was 5.7 ± 4.1 years with a range from 0.6 to 14.3 years. In this time frame, 11 patients (32.4%) were affected by oral cancer, developing a total of 15 carcinomas (three patients suffered two primary de novo malignancies). Among these oral cancers: nine were conventional squamous cell carcinomas, three were verrucous carcinomas, and three were in situ carcinomas. The mean age of malignant transformation was 67.2 ± 12.9, particularly 8 ± 8.5 from the onset of the lesions.

**FIGURE 3:**
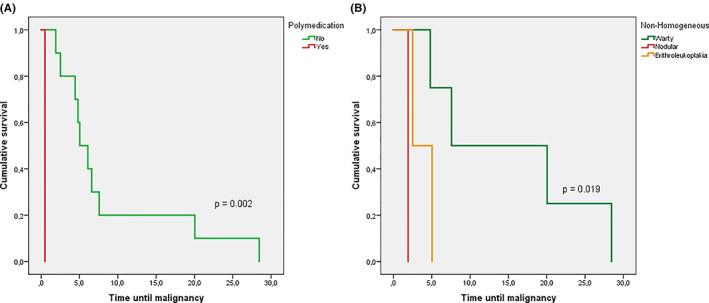
Kaplan–Meier malignant transformation curves according to the polypharmacy use (A) and clinical form (B)

In terms of risk factors associated with malignant transformation, epithelial dysplasia was highlighted. Both grading systems showed relevant usefulness to predict malignant transformation (Table 3). The results were inconclusive on whether a system was superior to others at predicting malignant transformation. Kaplan–Meier curves for malignant transformation showed that warty forms presented a higher mean estimate (15.2 years, 95% confidence interval [CI] 4.4–26 years) than nodular forms (1.9 years, 95% CI 1.9–1.9) (log‐rank *p* = 0.019). Likewise, polymedicated patients displayed an earlier malignant transformation (0.6 years, 95% CI 0.6–0.6) than non‐drug users (8.7 years, 95% CI 3.4–14.1) (log‐rank *p* = 0.002) (Figure 3).

**TABLE 3:** Relationship between the degree of dysplasia at the different times of follow‐up (T0‐T1‐T2‐TF) and malignant transformation

Variable	T0		T1		T2		TF	
	Malignant transformation		Malignant transformation		Malignant transformation		Malignant transformation	
	Yes *N* (%)	No *N* (%)	Yes *N* (%)	No *N* (%)	Yes *N* (%)	No *N* (%)	Yes *N* (%)	No *N* (%)
WHO grade system								
No dysplasia	6 (24%)	19 (76%)	1 (9.1%)	10 (90.9%)	0	3 (100%)	1 (10%)	9 (90%)
Mild dysplasia	1 (80%)	4 (20%)	2 (100%)	0	1 (33.3%)	2 (66.7%)	3 (42.9%)	4 (57.1%)
Moderate dysplasia	1 (100%)	0	1 (100%)	0	2 (100%)	0	1 (100%)	0
Severe dysplasia	0	0	0	0	0	0	0	0
Carcinoma in situ	1 (100%)	0	0	0	1 (100%)	0	1 (100%)	0
Carcinoma	2 (100%)	0	2 (100%)	0	2 (100%)	0	5 (100%)	0
Total	11 (32.4%)	23 (67.6%)	6 (37.5%)	10 (62.5%)	6 (54.4%)	5 (45.5%)	11 (45.8%)	13 (54.2%)
*p*	**0.05**		**0.007**		**0.08**		**0.009**	
Binary system								
No dysplasia	6 (24%)	19 (76%)	1 (9.1%)	10 (90.9%)	0	3 (100%)	1 (10%)	9 (90%)
Low‐grade dysplasia	2 (33.3%)	4 (66.7%)	3 (100%)	0	4 (66.7%)	2 (33.3%)	3 (42.9%)	4 (57.1%)
High‐grade dysplasia	1 (100%)	0	0	0	1 (100%)	0	1 (100%)	0
Carcinoma	2 (100%)	0	2 (100%)	0	1 (100%)	0	6 (100%)	0
Total	11 (32.4%)	23 (67.6%)	6 (37.5%)	10 (62.5%)	6 (54.4%)	5 (45.5%)	11 (45.8%)	13 (54.2%)
***p***	**0.07**		**0.002**		0.132		**0.004**	

*Note*: Significant results are presented in bold.

In relation to the lichenoid areas in the PVL lesions, of the 15 patients, only one suffered MT (6.6%), whilst those who did not present lichenoid areas (*n* = 19), underwent an MT in nine cases (52.6%) (*p* = 0.004). By means of binomial logistic regression, it was determined that the risk of malignancy was lower in patients in which PVL onset was associated with initial lichenoid areas. The response variables were nominal (no natural ordering in the outcome). The dependent variable was made up of two levels, the presence of lichenoid morphology or its absence among participants. The independent variable had two levels (i.e., malignant transformation or its absence). The reference category was the presence of lichenoid morphology in PVL patients on the basis of post hoc analysis. Thus, patients with an initial PVL lesion suffered an extreme higher risk of malignancy, particularly 15.55 times (95% CI 1.69–143.17; *p* = 0.015) than those who did present a lichenoid area.

## 4 DISCUSSION

Proliferative verrucous leukoplakia is a scarce and distinct clinical form of oral leukoplakia, characterized by its dynamic clinical course, continuous histopathological variations, and its potential to undergo a malignant transformation.^2^ The initial description of PVL provided by Hansen et al.^4^ more than 30 years ago is not easy to reproduce in clinical practice. In this vein, the diagnosis is often made late during the course of PVL, when the white patches are widespread, by which time, it is especially refractory to treatment.^18^ So, several authors have tried to adjust PVL diagnosis criteria to confront this problem.^5^

In general, the results of the present Spain study of PVL confirm observations from previous studies in the USA, Europe, and Asia.^4^, ^6^, ^11^, ^19^, ^20^ Regarding clinical location, Gandolfo et al.^19^ observed PVL lesions most frequently on the alveolar ridge/gingiva (41/47 [87.2%]). Bagan et al.^11^ also verified that the gingiva was the most frequent location of PVL in a series of 55 cases, affecting 89.1% of patients. In our series, gingiva was the most frequent location, we found that 31.8% PVL lesions involved this region. In this vein, the initial involvement of attached mucosa may play a pivotal role in early diagnosis and control of PVL.

We also verified the typical multifocal presentation of this disorder. The multifocal presentation of PVL and its risk of multiple primary oral carcinomas have been associated with the concept of field cancerization originated in 1953 from the histopathological observations of Slaughter and colleagues.^21^ This concept was introduced to describe the occurrence of multifocal neoplastic lesions in the oral epithelium, particularly in the adjacent non‐tumor epithelia of oral carcinomas.

Potentiating factors, such as smoking or alcohol intake, have not demonstrated any evident influence on either occurrence or progression in the present study. It is also worth mentioning that the consumption of these carcinogens was anecdotal in our study. This is consistent with previous case series addressing this issue.^2^, ^10^ The consumption of tobacco and alcohol does not seem to play an important role in the onset of this lesion This fact differs particularly from classical OL, for which tobacco is recognized as the main risk factor.^22^ Borgna et al.^20^ found no significant differences regarding smoking and alcohol use between 23 patients with PVL who progressed to malignancy and the 25 individuals with PVL without progression.

In agreement with studies of other predominantly Caucasian patient groups, PVL seemed to develop in late life and being more frequent in females without unhealthy habits.^23^ In terms of gender, although females were more commonly affected by PVL than males, no association between gender and malignant transformation was noticed. A recent meta‐analysis found that women have a statistically higher incidence of PVL, and an also increased risk for malignant transformation than males.^24^ Moreover, the mean age of patients was higher than 60 years as in most published studies; however, no clear association between age and malignancy was identified in the present cohort which is consistent with the previous body of literature.^10^

Moreover, this study reinforces the previously published evidence on the appearance of early lichenoid areas as helpful tool for diagnosis in the initial stages of PVL.^25^ This information is consistent with two previous studies conducted in England.^26^, ^27^ In a careful follow‐up study by McParland et al.,^27^ a transformation rate of 21.5% was reported in a group of 51 patients with PVL. They also ascertained that lichenoid areas were present in 22 patients. During follow‐up of 145 patients, Thomson et al.^26^ have elegantly demonstrated that one‐third of patients of its OPMDs cohort showed features both of oral lichenoid lesions and PVL, so they consider the hitherto cases as a disease presentation continuum. In the present study, we verified this clinical reality in patients affected by PVL and, what is even more important, we found that the persistence of lichenoid features in these lesions was a sign of better biological behavior or at least a valuable sign for early diagnosis. In this sense, PVL cases with initial lichenoid areas seem to create a subgroup of interest in terms of malignant transformation. This clinical scenario seems correct bearing in mind the malignant transformation rates of oral lichenoid lesions/oral lichen planus and “pure” PVL, which are considerably lower and higher, respectively, than those of these subsets of lesions.^28^, ^29^ This finding is very relevant since oral carcinoma develops in a high proportion of patients with PVL without a history of smoking, alcohol use, or both, that could influence the malignant transformation.^30^ For these reasons, the identification of subsets of patients at high risk who are in particular need of follow‐up or treatment remains a challenge to the clinician. From a histopathological point of view, the mutual appearance of these OPMDs may be feasible, since in early phases of PVL similar to in the lichenoid spectrum, the presence of lymphocytic infiltrates in the immediate sub‐epithelial region has been described.^8^, ^12^

Clinically, oral lichen planus (OLP) and oral lichenoid lesions (OLLs) are the main OPMD types that involve the appearance of lichenoid areas. Both disorders are also well known for its dynamic clinical presentation. In fact, cohort studies have shown that half of the patients affected by these diseases reflect this clinical reality.^31^ In this vein, these disorders are characterized for their relapses and remissions, their presentation as white reticular lesions, accompanied or not by atrophic, erosive, and/or plaque‐type areas. Bearing in mind this last subtype (i.e., OLP or OLLs showing plaque‐type areas), some authors have described the development of multiple widespread leukoplakias in patients previously diagnosed simply as pathologies inside the lichenoid spectrum.^32^ Chainani et al.^33^ described the same progression to OL in a cohort of patients initially diagnosed as OLP. Prompted by discussed literature, Gilligan et al.^25^ hypothesized that a subset of PVL cases could be presented as the evolution of OLP or OLLs but also could be a continuum of the same precancerous condition in the context of field cancerization. In the experience of the authors (P.G.V., A.B.C., and M.P.S.), the presence of a striae foci in the context of an oral widespread white lesion may lead clinicians to misdiagnose PVL as OLP or OLLs. This entails serious consequences since the clinical management of these entities has to be much stricter in the case of PVL to avoid delayed diagnosis and improve disease control. In our opinion, this assumption informs that patients affected by PVL must be monitored by specialists in oral medicine instead of general dental practitioners. This assertion may be in a part the reason which justified a single mouth neoplasm for a patient showing early lichenoid areas in the present longitudinal study.

Eleven patients in our series (32.4%) developed a carcinoma during follow‐up. Of the 11 carcinomas diagnosed in our cohort, all were T1/T2. None of the patients had lymph node involvement, and they were all alive and free of disease at the end of the follow‐up. In the experience of the authors, patients with PVL should be followed up at least once every 3 months and should be given exhaustive information on the possibility of malignant transformation of their lesions to achieve a desirable clinical follow‐up.

The findings of this study have to be seen in light of some limitations. First, the number of cases reported is small, the study is retrospective in design, and from one single unit. Second, given that it may take more than 10 years to undergo malignant transformation, there are some patients who may develop oral cancer in the future but have not undergone a malignant development at the moment. Third, data regarding the risk factors that may exert an influence on malignant transformation is too small to guarantee generalizations from the present study. Lastly, our clinical data registry could not ascertain the initial diagnosis of patients with lichenoid areas as canonical OLP or OLLs.

## 5 CONCLUSIONS

In summary, while this retrospective study has some limitations, it strongly highlights the high malignant transformation rate of PVL and the benefits of individual, regular follow‐ups for patients affected by this disorder. What can be delineated in the present study is that lesions with lichenoid areas/morphology outlined here are an initial stage through which PVL may start. Such information plays a pivotal role in the early diagnosis and control of this disorder. Further studies are needed to understand the presence of lichenoid areas in early stages of proliferative verrucous leukoplakia.

## AUTHOR CONTRIBUTIONS

**Catalina Barba‐Montero**: Conceptualization; formal analysis; investigation; methodology; validation; writing‐original draft. **Alejandro Ismael Lorenzo‐Pouso**: Conceptualization; data curation; formal analysis; investigation; methodology; project administration; resources; software; validation; visualization; writing‐original draft; writing‐review & editing. **Pilar Gándara‐Vila**: Conceptualization; data curation; formal analysis; funding acquisition; investigation; methodology; project administration; resources; software; supervision; validation; visualization; writing‐review & editing. **Andrés Blanco‐Carrión**: Data curation; investigation; methodology; supervision; validation; writing‐review & editing. **Xabier Marichalar‐Mendía**: Formal analysis; investigation; methodology; supervision; validation; visualization; writing‐review & editing. **Abel García‐García**: Conceptualization; data curation; funding acquisition; investigation; methodology; project administration; supervision; writing‐review & editing. **Mario Pérez‐Sayáns**: Conceptualization; data curation; formal analysis; funding acquisition; investigation; methodology; project administration; resources; software; supervision; validation; visualization; writing‐review & editing.

## CONFLICT OF INTEREST

The authors declare no conflict of interest.

### PEER REVIEW

The peer review history for this article is available at https://publons.com/publon/10.1111/jop.13317.

## INFORMED CONSENT

Written Informed consent was obtained for all patients participating in the study.

## Data Availability

The data that support the findings of this study are available from the corresponding author upon reasonable request.
